# Plant-Based Drugs and Vaccines for COVID-19

**DOI:** 10.3390/vaccines9010015

**Published:** 2020-12-30

**Authors:** Nasir Mahmood, Sarah Bushra Nasir, Kathleen Hefferon

**Affiliations:** 1 Department of Cell and Systems Biology, University of Toronto, Toronto, ON M5S 2E8, Canada; nasir.sbs1@gmail.com; 2 Department of Biochemistry, University of Health Sciences, Lahore 54600, Pakistan; 3 Forest Ridge Health Care Inc., Toronto, ON M5J 2V1, Canada; 4 Department of Life Sciences, Abdus Salam School of Sciences, Nusrat Jahan College, Chenab Nagar 35460, Pakistan; sarahbushra1@gmail.com

**Keywords:** coronavirus, antibodies, plants, vaccines, antivirals

## Abstract

The coronavirus SARS-CoV-2 has turned our own health and the world economy upside down. While several vaccine candidates are currently under development, antivirals with the potential to limit virus transmission or block infection are also being explored. Plant production platforms are being used to generate vaccines and antiviral proteins inexpensively and at mass scale. The following review discusses the biology and origins of the current coronavirus pandemic, and describes some of the conventional, synthetic, and plant-based approaches to address the challenge that it presents to our way of life.

## 1. Introduction

**Figure 1:**
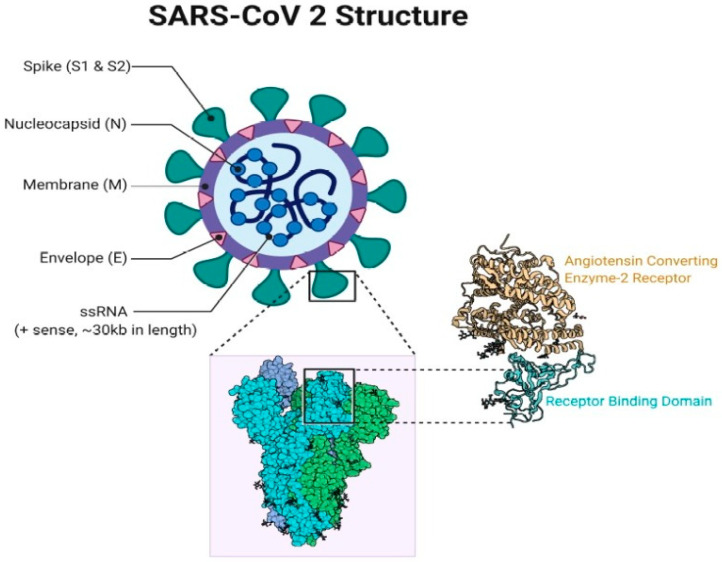
SARS-CoV-2 structure and its affinity with human ACE2 receptor (adopted from [15]., 2020).

Coronaviruses (CoVs) constitute a very diverse group of positive-sense enveloped RNA viruses with genomes ranging between 27–32 kb [1]. The viral structure comprises four structural proteins including the spike, nucleocapsid, membrane and envelope protein [2], several non-structural proteins and proteins derived from the host cell [1]. The CoVs display a characteristic ‘crown-like’ appearance under an electron microscope owing to the presence of club-shaped protein projections on the viral surface (ECDC, 2020; [3,4]). The simplified SARS-CoV-2 structure and its affinity with human angiotensin converting enzyme-2 receptor is illustrated in Figure 1. 

Coronaviruses belong to the order Nidovirales, which is classified into four families, including the Coronaviridae, Mesoniviridae, Arteriviridae and Roniviridae. Coronaviridae is categorized into two sub-families: the Coronavirinae and Torovirinae. The sub-family Coronavirinae is further divided into four genera based on serological and phylogenetic findings: *Alphacoronaviruses*, *Betacoronaviruses*, *Gammacoronaviruses*, and *Deltacoronaviruses* [5]. The Alphacoronaviruses and Betacoronaviruses are infectious in mammals only while Gammacoronaviruses and Deltacoronaviruses are infectious in birds with some viruses infecting mammals as well [6]. In the 1960s, coronaviruses were discovered in humans for the first time [7]. The earliest studied human viruses were the human coronavirus OC43 and 229E, both caused the common cold [8]. Coronaviruses also consist of emerging viruses such as the severe acute respiratory syndrome-related CoV (SARS-CoV) of Southern China (2003), the Middle East respiratory syndrome-related CoV (MERS-CoV) of Saudi Arabia (2012) [9,10] and SARS-CoV-2, the recently identified coronavirus in the Wuhan City of China (December, 2019) [11,12]. Among the coronaviruses known to infect humans, MERS-CoV, SARS-CoV and SARS-CoV-2 have the potential to cause severe disease while OC43, 229E, NL63, and HKU1 display mild symptoms [13]. SARS-CoV-2 is seventh among the coronaviruses that infect humans [14]. 

## 2. The COVID-19 Pandemic

A novel Coronavirus that recently emerged in Wuhan City, China was initially detected on 29 December, 2019, from four human cases who were all linked to the Huanan seafood market in Southern China. Patients displayed “pneumonia of unknown etiology” similar to the 2003 SARS. Deep sequencing of samples obtained from the patients’ lower respiratory tract revealed a novel strain of coronavirus that was named as severe acute respiratory syndrome coronavirus 2 (SARS-CoV-2) [16,17]. 

Scientists quickly identified the causative agent as betacoronavirus [12,18,19]. Phylogenetic analysis revealed that a viral genome consisting of 29,903 nucleotides shared 89.1% nucleotide similarity with SARS-like coronaviruses [18,20] previously detected in bats in China [21]. Although the virus has similarities with MERS-CoV and SARS-CoV, it is completely unique [11,12]. It was initially thought that the new virus may have lesser severity as compared to MERS-CoV and SARS-CoV. However, further evidence of a rapid upsurge in incidence and interpersonal transmission indicated that it is highly contagious [22,23,24,25,26]. The World Health Organization officially declared the outbreak caused by 2019-nCoV a pandemic on 11 March, 2020 [27] and termed the disease ‘COVID-19’ [11,12]. 

The SARS-CoV-2 virus particle has a spherical shape that exhibits some degree of pleomorphism with a diameter ranging from 60 to 140 nm, and distinct spikes 8–12 nm long [12]. SARS-CoV-2 seems well suited to bind the ACE2 human receptor with its spike protein having a polybasic (furin) functional cleavage site that favours the attachment of 12 nucleotides at the S1–S2 boundary [28]. The receptor-binding domain (RBD) of the spike protein is the most variable portion of the CoV genome [12,18] Six amino acids in the RBD are critical for attachment to the ACE2 receptors as well as determination of host range of viruses like SARS-CoV [29]. 

**Figure 2:**
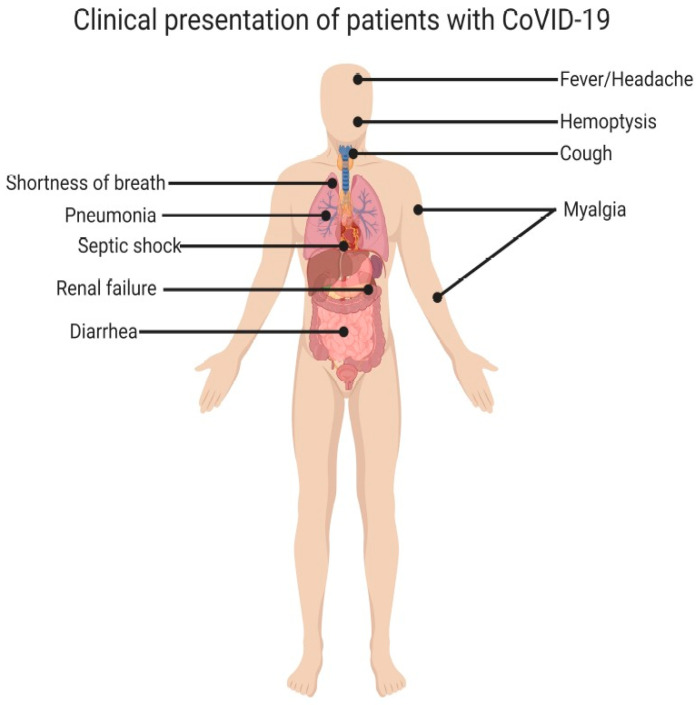
Clinical symptoms of COVID-19 patients (adopted from [15]).

The virus primarily spreads through the respiratory secretions of an infected person when he sneezes, coughs or talks [30]. Droplets of the infected person can infect others when they come in contact with their mucous membranes. Droplets are typically present within a range of 2 metres (6 feet) from the infected individual and do not last long in the air [30]. Infected surfaces can serve as a source of infection too if touched with the hand followed by touching the mouth, nose, and eyes. Symptomatic patients are believed to be most contagious [31]. The exact time required for virus incubation in the host is not known. However, it is thought to vary between 2 and 14 days post exposure to the virus, with five days being the most common time frame [31,32,33]. The age groups most affected are the middle aged and the elderly. Children do not appear to display symptomatic infection and, if they do, it is generally mild [34]. A study conducted on 1099 COVID-19 patients in Wuhan, China revealed the following common clinical features at the disease onset: fever (88%), fatigue (38%), dry cough (67%), myalgias (14.9%) and dyspnea (18.7%). Pneumonia was found to be the most severe consequence of the infection. In patients with pneumonia, breathing difficulties developed after an average of five days [33]. The clinical symptoms of COVID-19 patients are illustrated in Figure 2.

Important similarities exist between the symptoms of COVID-19 and previous betacoronavirus infections including fever, dyspnea, dry cough and ground-glass opacity in CT scans of the chest [35]. However, COVID-19 displayed some unique symptoms including sneezing, rhinorrhea and sore throat, indicating that the lower airway is being targeted. Moreover, chest radiographs of some cases upon admission indicated infiltrate in the lung’s upper lobe that is responsible for dyspnea associated with hypoxemia [36].

## 3. Vaccines and Antibodies under Clinical Trials

There is a lack of precise antiviral treatment and vaccines for COVID-19 currently [37]. However different potential vaccines and antibodies in different parts of the world are under trial, but we cannot say with certainty which vaccine and antibody will be successful and which will fail until/unless we have tested these vaccines and antibodies on a large human population. Future clinical trials of these vaccines and antibodies will determine their clinical efficacy. If a vaccine becomes available for COVID-19, vaccination rate has remained an issue of concern which requires proof such as “clear and convincing evidence, “beyond a reasonable doubt,” and “preponderance of the evidence” on the safety and effectiveness of the vaccine. Several vaccines and drugs are under trial against COVID-19 [38]. Table 1 below shows information about some of the vaccines and antibodies currently under clinical trials.

## 4. Potential Drug Therapies Against COVID-19

### 4.1. Remdesivir

Remdesivir is currently recognized as a proficient antiviral drug with promising potential against a broad range of RNA virus infections (including SARS/MERS-CoV) in cell cultures, nonhuman primate (NHP) models, and mice. Clinical development of Remdesivir to treat Ebola virus infections is underway [60]. Remdesivir is an analogue of adenosine which integrates into the emerging chains of viral RNA and marks their pre-mature termination showed that remdesivir was functional at the post entry stage of SARS-CoV-2 virus which supports its antiviral mode of action, acting as a nucleotide analogue. In a NHP model, demonstrated that an intravenous dose of 10 mg/kg of remdesivir conferred 100% resistance to Ebola virus infection with concomitant sustained levels (10 μM) in the blood. [61] revealed that EC90 value of this drug against SARS-CoV-2 in Vero E6 cells was found to be 1.76 μM, signifying its potential working concentration to be attained in NHP. Similarly, Remdesivir was also found to inhibit SARS-CoV-2 infection in a human cell line (liver cancer Huh-7 cells). [62] studied the effect of remdesivir on 53 hospitalized COVID-19 patients. Remdesivir was administered for 10 days with 200 mg on the first day and 100 mg on the other nine days. Results showed improvement in 36 out of 53 patients demonstrating an efficacy of 68%. However the study lacked a control group, therefore the results remain inconclusive. More recently, WHO has issued a conditional recommendation restricting the usage of remdesivir in hospitalized individuals irrespective of disease severity since there is no current evidence that remdesivir promotes survival and recovery in patients. 

### 4.2. Favipiravir

Favipiravir is a pyrazine carboxamide derivative anti-viral drug that has been approved to treat influenza in Japan. It is a prodrug, intracellularly phosphorylated and ribosylated to give rise to the active favipiravir ibofuranosyl-5′-triphosphate (T-705-RTP) metabolite [63]. T-705-RTP hinders viral replication by competing with purine nucleosides and becoming incorporated into the viral RNA and thereby constraining the viral RNA dependent RNA polymerase (RdRp) [64]. Apart from the inhibitory effect on influenza virus, Favipiravir displays inhibition of a broad variety of RNA viruses including bunyavirus, filoviruses, falvivirus, and arenavirus that cause hemorrhagic fever [63]. During the Ebola outbreak in 2014–15, favipiravir showed improved survival rates in patients tested in Guinea. A retrospective study on Ebola virus disease found that patients who were treated with favipiravir in addition to WHO-recommended supportive treatment showed an overall higher survival rate and time and a >100-fold reduction in viral load [65]. Genome sequencing of SARS-CoV-2 revealed that it has the same *RdRp* gene as found in SARS-CoV and MERS-CoV [12,66,67]. Therefore, favipiravir can be considered for use against COVID-19 but recognized in vitro and animal studies are not yet available. A clinical trial to check the efficacy and safety of favipiravir was carried out on 80 patients in Shenzhen [68]. Results demonstrated that the viral clearance time was shorter in 35 patients who received favipiravir as compared to 45 patients who served as controls. Moreover, X-ray studies confirmed that there was a higher improvement rate in chest imaging of the favipiravir group as compared to the control group (91.43% vs. 62%) [68] Another randomized clinical trial of favipiravir on COVID-19 patients demonstrated an effective control with increase in seven days clinical recovery period from 55.86% to 71.43% [69].

### 4.3. Lopinavir/Ritonavir

A study carried out by Jian-ya et al. [70] on 51 patients of COVID-19 with interferon, Ritonavir, traditional medicine and Lopinavir and corticosteroids (3–5 days) resulted in successful recovery of 50 patients. Another study by Qin et al. showed that treatment with lopinavir along with interferon and moxifloxacin to non-ICU patients and administration of methylprednisolone in addition to the above drugs to treat patients in intensive care unit (ICU) resulted in the discharge of 26 patients from ICU and 16 patients from hospital [71]. Case reports on the successful treatment of COVID-19 patients with Lopinavir/Ritonavir includes a 54 year old patient who received two tablets of Lopinavir (200 mg)/Ritonavir (50 mg), 12 h apart, after 10 days of illness and resulted in the reduction of viral load which gradually became negligible. However, a recent randomized, controlled trial by Cao et al. [72] on 199 COVID-19 positive patients with Lopinavir–ritonavir resulted in no obvious differences between the standard care group and the treatment group which suggests that future trials are needed to exclude or confirm its efficacy.

### 4.4. Convalescent Plasma (CP) Therapy 

Convalescent plasma (CP) therapy is a classic approach of immunotherapy that has been in use for over a century to treat various infectious diseases. CP proved successful in treating SARS, MERS and pandemic 2009 H1N1 over the last two decades with satisfactory results [61,73,74,75]. A meta-analysis of 32 studies involving infection caused by SARS coronavirus and influenza indicated a significant statistical pooled odds reduction in mortality with CP therapy as compared to placebo and non-treatment groups with odds ratio of 0.25%, confidence interval 95%, 0.14–0.45) [76].

A recent study by Shen et al. [77] was carried out on five critically sick COVID-19 patients who were receiving aided breathing through ventilators and along with antiviral therapy and methylprednisolone. Plasma transfusion to these patients resulted in normalization of body temperature within three days in four out of five patients, reduction in the Sequential Organ Failure Assessment (SOFA) score, increase in PAO2/FIO2 and decrease in viral load within 12 days. However, the small sample size precludes a conclusive statement about the efficacy of CP therapy. Duan et al. [78] conducted a study on 10 critical COVID-19 patients who were receiving antiviral agents and supportive care. A 200 mL dose of CP with above 1:640 neutralizing antibody titers obtained from recovered donors was transfused to these patients. CP transfusion resulted in increased neutralizing antibody levels in five patients with four patients maintaining a high neutralizing antibody ratio (1:640). A significant improvement in clinical symptoms without any adverse effects was observed including a rise in lymphocyte count, improvement in lung lesions, reduced C-reactive protein and increase in oxyhaemoglobin saturation within three days. However, further investigations regarding the optimal dose, time point and clinical benefits are required.

## 5. Herbal Therapies Against COVID-19 

The application of phytomedicines has increased due to their therapeutic value when compared to allopathic medicines as these bio-compounds exhibit fewer side effects [79]. The possibility of using plant-derived phytochemicals (particularly polyphenols) with putative active substances (e.g., flavonoids, gallates, and quercetins), which are potent agents prohibiting the proliferation of the COVID-19-inducing coronavirus has recently been reviewed by [80]. They can be used as pharmaceutical preparations or functional foods. Herbal plants such as *Artemisia kermanensis*, *Eucalyptus caesia*, *Mentha* spp., *Rosmarinus officinalis*, *Satureja hortensis*, *Thymus* spp., and *Zataria multiflora* are typical examples of rich sources of phenolic compound [81,82]. Biflavonoids from *Torreya nucifera* inhibited the replication of SARS-CoV 3CLpro [83].

*Senna* L. is large genus of flowering plants in the legume family (Fabaceae) of subfamily Caesalpinioideae, comprised of 300–350 species [84]. It is a widespread and diverse genus. Many species of *Senna* are commonly used in foods and herbal medicine [85]. In different parts of the world, the whole *Senna alata* plant is currently being used in the treatment of flu, fever, malaria, and large number of other medical conditions due to the presence of bioactive compounds in the plant, including quinones, alkaloids, and terpenes [86,87,88,89,90]. The leaf extract of *Senna alata* considerably inhibited 3D7 strain of the *Plasmodium falciparum* parasite in vitro [87,88]. Recently, it was determined that use of an aqueous extract obtained after boiling of 5 g of *Senna* leaves in 500 mL water for 10 min provided relief from virus symptoms in COVID-19 patients (unpublished data). After boiling, it is recommended that half of the aqueous leaf extract can be used immediately while the second half can be used after 8–10 h to avoid toxicity. In the intervening time, water taken frequently can avoid toxicity. In order to avoid the bitter taste of aqueous leaf extract, honey can be added, which has also an antimicrobial effect. As *Senna alata* plants are currently being used to treat different medical conditions in different parts of world, including Africa and Asia, there is a need to confirm *Senna* leaf aqueous extracts in COVID-19 patients as a herbal therapy. 

Lianhuaqingwen (LH), a Traditional Chinese Medicine formula composed of a combination of 13 herbs was shown to suppress SARS-CoV-2 replication, reduced pro-inflammatory cytokine production, and changed the morphology of SARS-CoV-2 cells [90]. These herbs are presented in Table 2. [91] demonstrated that LH also has a comparable antiviral potency against the SARS-CoV-2 virus in vitro. It was noteworthy that transmission electron microscopy (TEM) revealed that the number of virus particles in infected patients was greatly reduced in cells infected with SARS-CoV-2 that were treated with LH at 600 µg/mL. LH is widely used for a variety of respiratory virus infections, including influenza virus. The precise mechanism of action of LH to reduce virus infection remains unknown, although it has been shown to also reduce cytokine release from infected cells, suggesting that multiple levels of action are taking place. 

Chinese health authorities in 23 out of 31 provinces have issued herbal remediy programs to prevent the spread of COVID-19. The top two herbal formulas used were *Radix astragali* (Huangqi) and *Glycyrrhizae radix* Et Rhizoma (Gancao) [92]. Another study recommends the use of tender leaf of *Toona sinensis* Roem, a popular Chinese vegetable that is already used safely. In vitro studies show that the tender leaf of *Toona sinensis* Roem, can inhibit SARS-CoV [93]. It is expected to also block SARS-CoV-2.

As mentioned above, licorice root has some bioactive properties, including antitumoral, antiinflammatory, and antiviral effects on health. Glycyrrhizic acid (glycyrrhizin, GL) and its aglycone glycyrrhetinic acid (GLA) are active against a broad spectrum of viruses, including herpes viruses, flaviviruses, Hepatitis C virus, human immunodeficiency virus, and SARS coronavirus (SARS-CoV) in vitro. Virus inhibition has been demonstrated in Vero cells and in patients [35,94,95,96]. Further dissection of glycyrrhizic acid indicated that sugar moieties are responsible for the anti-SARS activity, as a replacement of these with functional groups resulted in a loss in activity.

Earlier studies using herbs to block SARS could also successfully inhibit SARS-CoV2 infection. For example, Wen et al., 2011 [97] examined extracts of over 50 traditional Chinese medicinal (TCM) herbs on anti-SARS-CoV activity using a Vero E6 cell-based cytopathogenic effect (CPE) assay. The authors were able to demonstrate that six novel herbal extracts may be used as potential SARS drug targets. The herbal extracts were derived from *Rhizoma cibotii* (gǒu jǐ), *Gentianae radix* (lóng dǎn), *Dioscoreae rhizoma* (shān yào), *Cassiae semen* (jué míng zǐ), and *Loranthi ramus* (sāng jì shēng); all of them inhibited SARS-CoV replication, and two inhibited virus protease activity.

Studies using phlorotannins isolated from the edible brown algae *Ecklonia cava* found that several of these bioactive compounds were able to inhibit SARS-CoV activity by functioning as protease inhibitors. One of these, dieckol, had the most potent antiviral activity; this took place through competitive binding at the catalytic site of the protease [98]. It is feasible that dieckol would also have an inhibitory effect against SARS-2.

## 6. Plant Molecular Pharming to Combat COVID-19

Plants are being used for the production of recombinant vaccines and drugs for more than 30 years and the whole process is described under term ‘molecular farming’ [99,100]. Secondary metabolites have significant biological and ecological functions in plants; particularly advantageous is their role in chemical defense because of their antioxidative and antimicrobial activities. Thus, molecular farming is used for the large-scale production of valuable secondary metabolites. In addition, metabolic engineering tools can be used to overwhelm the bioactive-compounds availability limitations from medicinal plants and to improve the productivity beneficial from both bioprocessing and molecular farming [101] The synthesis of desirable recombinant proteins (pharmaceuticals and industrial proteins) using whole plants or in vitro cultured plant tissues/cells in large-scale bioreactors is termed molecular farming. The advantages of plant-based reactors have been described in a review of molecular farming by Mohammadinejad et al. [101] as follows: (i) lower cost in maintenance; (ii) lower risks of contamination from animal pathogens; (iii) competence to implement modifications in eukaryotic post-translational machinery function; and (iv) being amenable to the large-scale manufacturing process.

**Figure 3:**
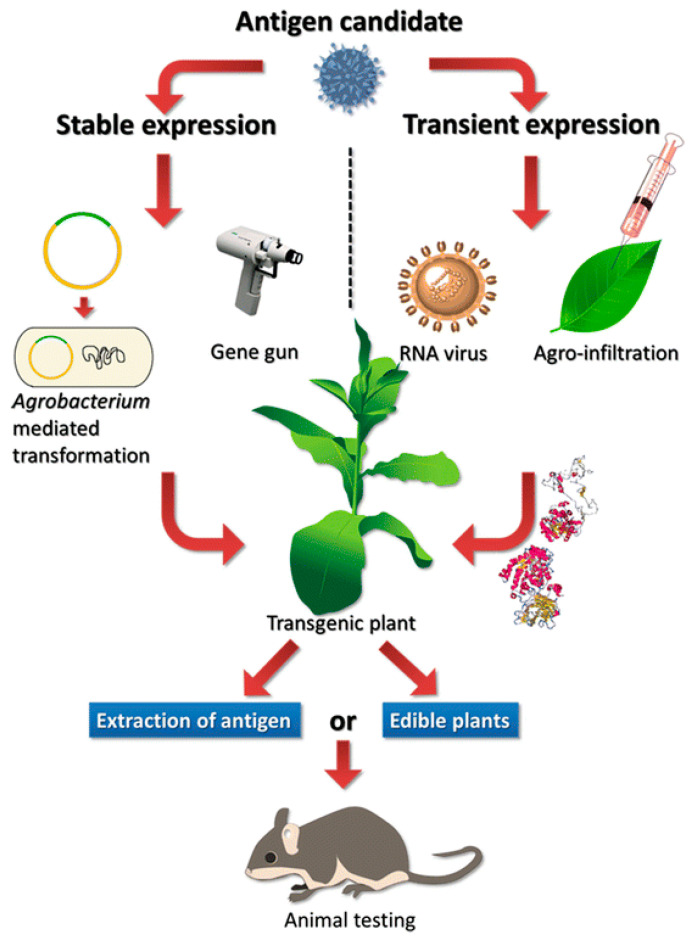
Schematic diagram of plant pharming.

Vaccines generated in plants have been shown to elicit a robust immune response in humans and animals (Figure 3). Plants have a great ability to act as a bioreactor system that supports many important biological processes including virus-like particles (VLPs) and vaccines. Transformation of plants with foreign genes leads to protein drugs, vaccines, and antibodies against different human pathogens hence plants make it easy to deal with safe, inexpensive, and provide trouble-free storage of protein vaccines and drugs [102]. Many research studies and clinical trials have shown that plant-made vaccines are safe and efficacious [103,104]. Examples of plant-made vaccines and therapeutics produced by molecular pharming include vaccines to combat cholera, Dengue fever virus and Hepatitis B virus, monoclonal antibodies to HIV and Ebola virus, and a therapeutic agent to provide glucocerebrosidase and help Gaucher Disease patients [104,105]. Several plant pharming companies and research labs have taken up the challenge to combat COVID-19. At the same time, there is a dramatic shortage of COVID-19 tests that could be alleviated by producing diagnostic agents in plants [106]. A few examples of vaccines, diagnostics for test kits and antiviral therapeutics are presented in the following section.

Medicago, a biopharmaceutical company based in Canada, has successfully developed a virus-like particle (VLP) of the coronavirus 20 days after obtaining the SARS-CoV-2 genetic sequence. Instead of using egg-based methods to develop vaccines, this technology inserts a genetic sequence encoding the spike protein of COVID-19 into *Agrobacterium*, a common soil bacterium that is taken up by plants [107]. The resulting plants that are developed produce a virus like particle that is composed of a plant lipid membrane and COVID-19 spike protein. Medicago is using the plant *Nicotiana benthamiana*, a close relative of the tobacco plant, to produce VLPs of the SARS-CoV2 virus (COVID-19: Medicago’s Development Programs). The VLPs are similar in size and shape to actual coronavirus but are lacking in nucleic acid and are, thus, noninfectious. Medicago has successfully completed its Phase 1 clinical trials and is currently working on Phase 2 clinical trials [108]. Previously, Medicago has made VLPs composed of influenza virus haemagglutinin, and have demonstrated their safety and efficacy in animal models as well as in human clinical trials [109]. The cost of producing a plant-made vaccine based on VLPs is a small fraction compared to its conventional counterpart [110].

In Canada, the University of Western Ontario and Suncor are developing diagnostic test kits for COVID-19 using algae as a production factory to make the viral spike proteins [111]. Algae has long been considered a potential platform for generating pharmaceutical proteins as well as industrial proteins, such as cellulases [112]. Algae is a superior biofactory alternative because it is easy to grow and can be readily modified to produce the viral proteins.

British American Tobacco, through its biotech subsidiary in the US, Kentucky BioProcessing (KBP), is developing a potential vaccine for COVID-19 and is currently undergoing pre-clinical testing [113]. Experts at KBP cloned a part of the genetic sequence of SARS-CoV-2, which they used to develop a potential antigen that was inserted into *Nicotiana benthamiana* plants for production. The vaccine has elicited a positive immune response by pre-clinical testing and will be onto Phase 1 human clinical trials soon [114]. BAT could manufacture as much as 1–3 million doses of COVID-19 vaccine per week (they made 10 million vaccines of flu in a month as well as an Ebola vaccine using the same plant-based approach) [115]. 

South African company Cape Bio Pharms (CBP) is also responding to the SARS-CoV-2 pandemic through the production of reagents in plants, which could be used for diagnostic kits [116]. CBP is producing SARS-CoV-2 Spike S1 reagents consisting of various regions of the glycoprotein attached to various fusion proteins. The company, based in Cape Town South Africa, is also collaborating with antibody manufacturers to produce antibodies against these proteins [116]. 

Another example of a plant molecular pharmed solution to COVID-19 is taking place in the department of nanoengineering at the University of California, San Diego. Researchers in Nicole Steinmetz’ lab have been using Cowpea mosaic virus like particles, with B- and T-cell epitopes from the S protein of SARS-CoV-2 displayed on their icosahedral surfaces [117]. The recombinant virus harboring these COVID-19 epitopes can be applied in the form of an implanted microneedle technology incorporating VLP vaccines to skin and will elicit an immune response to SARS-CoV-2 [118]. 

The Steinmetz research group have recently developed positive control probes, composed of Cowpea mosaic virus-like particles, to be used as COVID-19 diagnostics and improve the accuracy of COVID-19 tests. The researchers hope that these positive controls, which are stable at room temperature for prolonged periods of time and cheap to generate, could be useful in resource-poor settings [32]. 

Another collaboration between two research groups in Toronto, Canada, has brought about a novel way to fight COVID-19 using a synthetic peptide that binds to the viral deubiquitinase (DUB) and is carried by a plant virus. The work initially began by examining the role of the virus protease, located in ORF 1a of the coronavirus genome responsible for the related MERS virus. This protease contains a deubiquitinase activity as a means of protecting the virus from the innate immune system of the cell. Ubiquitin is a protein found in eukaryotic cells that play an important role in the regulation of proteins. It labels unwanted proteins (poorly folded proteins, viral proteins) to be degraded by the proteasome into shorter fragments or amino acids that can be recycled for cellular metabolism. Some viruses, such as coronavirus, express deubiquitinases (DUBs) to prevent destruction by the cell. 

**Figure 4:**
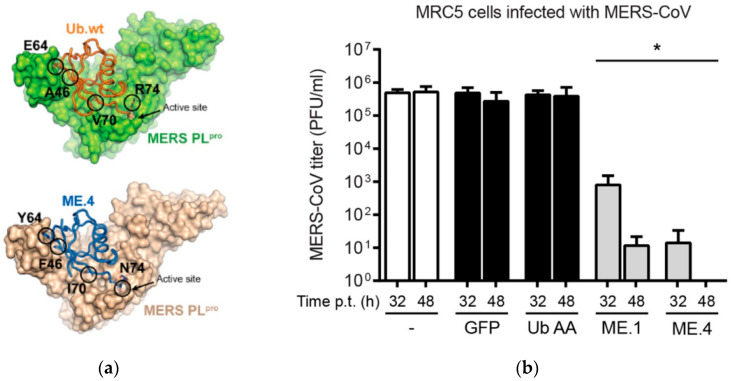
Synthetic ubiquitin variant (UbV) block MERS virus infection in a human cell line [119]. (**a**) MERS PLpro in complex with wild type ubiquitin or the inhibitory ubiquitin variant ME4. (**b**) UbV ME4 leads to drastic reduction in virus titer in MRC5 cells infected with MERS-CoV.7. Future Research against COVID-19.

A synthetic peptide of approximately 80 amino acids and known as a ubiquitin variant (UbV) was created by phage display library design and shown to bind tightly to MERS DUB at its ubiquitin binding site, thus blocking its deubiquitinase activity as well as its proteolytic activity (Figure 4). This synthetic UbV was shown to block MERS virus infection in a human cell line, using a lentivirus vector for cell entry [119]. An analog to this UbV, which selectively binds to the DUB of SARS-CoV2, has recently been engineered for use in the current pandemic [120]. Both MERS and COVID-19 UbVs have been fused to the N-terminus of the coat protein of a plant virus expression vector known as Papaya mosaic potexvirus (PaMV) (unpublished results). The UbV:CP fusion protein can assemble into virus like particles. PaMV has previously been shown to enter human cells via vimentin, a cytoskeletal protein. The virus nanoparticle, loaded with COVID-19 UbV, can enter cells and block virus infection. Potexvirus nanoparticles have been shown to successfully enter the epithelial cells of lungs when introduced in the form of an aerosol spray. It is possible that these VLPs can be loaded into an inhaler to treat the lungs of infected and uninfected patients. The ubiquitin variant is also being produced in a plant geminivirus vector, to be purified as an antiviral for COVID-19 patients (Manuscript in preparation). Geminiviruses, such as Bean yellow dwarf virus, have been engineered to produce large amounts of pharmaceutical proteins from plants in relatively short periods of time [121]. A novel synthetic antibody to COVID-19 that was engineered from a phage display library is also currently being examined using the geminivirus vector system [122]. 

The COVID-19 pandemic is a challenge for us all. As a result of the current COVID-19 pandemic, therapies such as Remdesivir, convalescent plasma (CP), and *Senna* leaf extracts are the best available against COVID-19, until we have vaccine candidates in hand which have successfully undergone laboratory experiments, animal trials, and all phases (1–3) of clinical trials. Possible targets including the spike, nucleocapsid, membrane, envelope, viral RNA polymerase, and 3-chymotrypsin-like protease (3CL^pro^), which cleaves the virus polyprotein at 11 distinct sites to generate various non-structural proteins that are important for viral replication, are all being used to develop potential vaccines and antiviral drugs. Virus like particles (VLPs) of SARS-CoV-2 may act as promising vaccines because they have the potential to activate the human immune response in a fashion similar to the original virus.

There is need to explore plant-based systems to check whether VLPs with retained structure and in sufficient quantity can be generated in these systems [123]. On the one hand, this can include the further refinement of herbal extracts, particularly ones that had been used in the past to successfully inhibit SARS-CoV, as they may also function to block SARS- CoV-2. The use of attenuated viruses and viral vectors in humans as vaccines may pose certain health risks involving the possibility of mutation (in the case of attenuated viruses) and recombination (in the case of viral vectors). The development of monoclonal antibodies against SARS-CoV-2 may also not be a long-term solution due to potential adverse reactions. Thus VLPs of SARS-CoV-2 generated by a plant expression system may act as a viable vaccine for the future.

## Figures and Tables

**Table 1:** Vaccines and antibodies currently under clinical trials for COVID-19.

Name of Vaccine	Nature	Company/Institution, Country	Reference
Recombinant subunit vaccine	It is a trimeric Spike-protein subunit vaccine expressed in mammalian cell system. The company claims to have preserved the original trimeric viral spike protein	Clover, China	[39,40]
ChAdOx1 nCoV-19	Weakened version of a common cold virus (adenovirus) in which Spike glycoprotein from SARS-CoV2 has been added	Oxford University, UK	[41,42]
mRNA-1273 vaccine	mRNA (messenger RNA) has been used to make this vaccine. It directs body cells to express a viral protein that would elicit an immune response. Promising results received in animals	Kaiser Permanente Washington Health Research Institute (KPWHRI), USA	[43]
Covigenix	It is a DNA-based vaccine that will directly induce a plasmid to encode the antigen against which an immune response is required. Phase 1 clinical trials underway	Entos Pharmaceuticals, Inc., Canada	[44]
Gimsilumab	It is based on Monoclonal antibodies that selectively inhibit granulocyte-macrophage colony-stimulating factor (GM-CSF). Phase 2 clinical trials underway	Roivant Sciences Ltd., USA	[45,46]
BNT162 vaccine	It is based on a nucleoside-modified RNA expressed in lipid nanoparticles that encodes the viral spike protein to elicit an immune response	Precision Vax LLC, Germany	[47]
Adcovid	It is based on the expression of receptor-binding domain (RBD) of the SARS-CoV-2 spike protein. Provides benefits of single dose efficacy, intranasal administration, and convenient storage conditions. Phase 1 clinical trials are underway	University of Alabama, UK	[48]
TJM2 vaccine	Consists of a neutralizing antibody that has a high affinity for human GM-CSF. Binding to GM-CSF results in inhibition of inflammatory responses to reduce disease complications. Phase 1 trials are underway	I-Mab Biopharma, China	[49]
Coronavirus-Like Particle COVID-19 vaccine (CoVLP)	VLPs of spike (S) glycoprotein of SARS-CoV2 have been produced in plant system. Phase 2 clinical trials are underway	Medicago, Canada	[50]
AT-100	Engineered version of a human recombinant protein that reduces inflammation and infection in the body	Airway Therapeutics Inc., USA	[51]
TZLS-501	Monoclonal antibody targeting the receptor for IL-6 to reduce cytokine storm and prevent exaggerated immune response.	UK/US combine company	[52]
INO-4800	It is a DNA based vaccine containing the plasmid pGX9501, which encodes the Spike glycoprotein of SARS-CoV-2.	INOVIO, China	[53]
Avian Coronavirus Infectious Bronchitis Virus (IBV) vaccine	By-product of the IBV vaccine, consists of a protein vector that secretes a soluble chimeric protein carrying the viral antigen into tissue resulting in production of antibodies	Migal Research Institute, Israel	[54]
TNX-1800	Live modified horsepox virus vaccine; consists of modified horsepox virus that expresses the SARS-CoV2 protein	Tonix Pharmaceuticals, USA	[55]
Vaxart’s coronavirus vaccine	The vaccine consists of a viral vector that carries genes for two SARS-CoV-2 proteins, the nucleocapsid and spike. The vector carries both genes to the cell along with a strong adjuvant that elicits an immune response to the viral proteins.	Vaxart, USA	[56,57]
Russian vaccine (Sputnik V)	It is an adenoviral-based vaccine that uses weakened virus to generate an immune response	The Gamaleya Center, Russia	[58,59]

**Table 2:** Herbs involved in Lianhuaqingwen, used in traditional Chinese medicine to suppress SARS-CoV-2 replication.

Common Name	Scientific Name	Chinese Name
Weeping forsythia	*Forsythia suspensa* (Thunb.) Vahl	Lián qiáo
Chinese ephedra	*Ephedra sinica* Stapf	Cǎo má huáng
Japanese honeysuckle	*Lonicera japonica* Thunb.	Rěndōng
Woad	*Isatis indigotica* Fortune	Sōng lán
Mint	*Mentha haplocalyx* Briq.	Bò hé
Thick-stemmed wood fern	*Dryopteris crassirhizoma* Nakai	Cū jīng lín máo jué
Golden Root	*Rhodiola rosea* L.	Hóng jǐng tiān
Gypsum	*Gypsum Fibrosum*	Shí gāo
Patchouli	*Pogostemon cablin* (Blanco) Benth.	Guǎng huò xiāng
Chinese Rhubarb	*Rheum palmatum* L.	Zhǎng yè dà huáng
Fish Mint	*Houttuynia cordata* Thunb.	Yú xīng cǎo
Licorice	*Glycyrrhiza uralensis* Fisch.	Gāncǎo
Siberian Apricot	*Armeniaca sibirica* (L.) Lam.	Shān xìng
